# Integrating Dental Professionals Into Aged Care With Focus on Australia: A Scoping Review

**DOI:** 10.1111/ger.12784

**Published:** 2025-01-12

**Authors:** Kelsey West, Julie Saunders, Linda Slack‐Smith

**Affiliations:** ^1^ School of Population and Global Health Perth Western Australia Australia

**Keywords:** aged care, dementia, oral health care, oral health therapist

## Abstract

**Objectives:**

To summarise the current evidence on the involvement of dental hygienists (DHs) in residential aged care facilities (RACFs) with respect to the feasibility of integration improved oral health for residents with dementia and multidisciplinary collaboration.

**Background:**

The oral health of RACF residents with dementia is reported to be poor. However, little is known about how DHs can be integrated into RACFs to improve oral health, particularly as part of a multidisciplinary team.

**Method:**

A scoping review was undertaken in accordance with the Joanna Briggs Institute (JBI) method. Multiple databases were searched for peer‐reviewed articles and grey literature that included a DH working in a RACF with dementia patients, or as part of a multidisciplinary team. Data were charted using a modified version of the JBI source of evidence template.

**Results:**

Fifty‐eight studies were identified for inclusion. Integration strategies were categorised as support‐focused or service‐focused, but there was little evaluation of their feasibility. Five key facilitators to multidisciplinary collaboration were identified: using multiple strategies; clearly defining roles; changes to existing administrative systems; fostering multidisciplinary collaboration skills; and encouraging innovation. However, no examples of collaboration within RACFs were identified.

**Conclusion:**

There has been limited effort in multidisciplinary collaboration or integration of DHs into RACFs with some evidence that both support‐focused and service‐focused strategies can improve the oral health of residents with dementia.

## 1 Introduction

The oral health of older adults living in residential aged care facilities (RACFs) is generally poor, especially those with dementia [1, 2, 3, 4, 5]. Many higher income countries are experiencing an ageing population [6], meaning that the prevalence of dementia is likely to rise also [7]. Simultaneously, older adults are retaining more of their natural teeth than previous generations [8]. The ‘consequence of success’ of improved oral health earlier in the life course is that older adults require more complex treatments and time‐consuming hygiene routines to maintain their natural teeth [9]. Many people in the later stages of dementia require assistance with oral hygiene routines [10] and are often resistant to the efforts of caregivers [11, 12, 13, 14]. Additionally, there are many barriers to providing dental services in RACFs, such as limited domiciliary services, negative attitudes from RACF staff combined with a lack of knowledge and training and a lack of financial incentives for dental professionals to work in RACFs and the wider public healthcare sector [14].

The World Health Organisation advocates for models of oral health care focused on disease prevention and health promotion and the integration of oral health services into primary care [15]. This entails greater collaboration between dental professionals and other health professionals to deliver a more holistic and patient‐centred approach to healthcare [15, 16, 17, 18, 19, 20]. However, many aspects of the current dental system do not reflect this ideal model, particularly in RACFs. Dental services are often provided *ad hoc*, with little structure, and dental professionals typically work in separation from the primary care team [5, 14, 21, 22]. This is despite the growing body of literature elucidating the important role oral health plays in maintaining good overall physical and mental wellbeing and the relationship between poor oral health and systemic diseases [23, 24, 25, 26, 27, 28, 29, 30, 31, 32, 33]. Evidence suggests a causal relationship between poor oral health and aspiration pneumonia [29, 33], two preventable conditions which are prevalent in older adults [22, 23].

One strategy to shift towards a disease prevention and health promotion model of care within RACFs and overcome barriers to accessing dental services is to integrate dental hygienists (DHs), dental therapists (DTs) and oral health therapists (OHTs) into RACFs to provide dental services [14, 34, 35, 36, 37, 38, 39]. Access to a dedicated oral health professional can improve the oral health of residents [40, 41, 42, 43].

In Australia, DHs, DTs and OHTs are important contributors to the oral health workforce. They are required to complete a program of study approved by the Dental Board of Australia and register with the Australian Health Practitioner Regulation Agency (AHPRA). Their role is typically oriented towards prevention and health promotion [44], with their scope of practice including preventive services and periodontal treatments excluding surgical interventions. They can provide services to both children and adults; however, some services for adults require additional training. Given the varied international definitions of DHs, DTs and OHTs, the authors use the term DH broadly to refer to all three professions.

DHs are also well‐suited to multidisciplinary collaboration. The authors use the term ‘multidisciplinary’ as a broad term that encompasses multidisciplinary, interdisciplinary and transdisciplinary health care providers [45]. Collaboration within a dental team is common, and the expanded registration standards and scope of practice granting them more autonomy enable them to explore roles outside of the traditional dental clinic practice model [46].Their success in exploring non‐traditional roles hinges on their ability to collaborate with other health professionals. Multidisciplinary collaboration is especially important in care for RACF residents with dementia, because they are often medically compromised with conditions becoming increasingly complex over time [47, 48] and require ongoing, individualised care involving many health professionals [49].

Despite the potential benefits of integrating dental professionals such as DHs into RACFs, relatively little attention has been paid to how integration may look in practice, and whether it is feasible [14, 21]. We also need to ensure that integration will benefit those most in need of affordable and accessible oral healthcare, such as RACF residents and especially residents with dementia, since they face additional barriers to accessing dental services and commonly have poor oral health. Additionally, the ‘silo’ mentality towards oral healthcare and general healthcare service provision has led to little multidisciplinary collaboration involving dental professionals, including in RACFs [3]. Hence, identifying strategies promoting multidisciplinary collaboration will assist in effectively integrating DHs into RACFs and their existing multidisciplinary teams. Therefore, the aims of this study were to map the current evidence pertaining to the research questions listed below.

1. Is the integration of DHs into RACFs feasible? If so, how might this look?
2. What strategies can be used to promote effective multidisciplinary collaboration between DHs and other health professionals in RACFs?
3. Can the integration of DHs into RACFs improve the oral health of residents with dementia?

We aim to report the types of evidence that address and inform practice in this field and identify gaps in the research. To achieve these aims, a scoping review was conducted in accordance with the Joanna Briggs Institute (JBI) method [50]. Because this is an emerging area of interest, a scoping review was chosen, as it is useful for examining emerging evidence when it is unclear what specific questions can be valuably addressed [50]. The JBI method guides authors in developing an a priori protocol with predefined objectives and methods. A formal assessment of the methodological quality is not usually performed in a scoping review. Rather, the primary focus of a scoping review is to summarise the currently available evidence regardless of quality [51]. A step‐by‐step guide to the JBI method of scoping reviews is included in the JBI Manual for Evidence Synthesis [50].

## 2 Methods

A review protocol was developed prior to conducting the scoping review. Given Prospero does not register scoping reviews, the protocol was not registered.

### 2.1 Inclusion Criteria

Inclusion criteria were developed using the ‘Population‐Concept‐Context (PCC)’ framework outlined by the JBI method of scoping reviews [50]. The PCC helps reviewers to identify the main focus and context of the scoping review questions, using this as a guide for the inclusion criteria [52]. Given the emerging nature of this field, the inclusion criteria were purposefully broad, to capture a wider breadth of literature.

### 2.2 Population

Similar terms for dementia (e.g., ‘cognitive impairment’) were accepted. Dementia status could be indicated by the researchers, RACF staff or by scores on standardised cognitive tests. Similar terms to RACF (e.g., ‘nursing home’) were accepted. As the OHT profession is unique to Australia, the current evidence base surrounding them is limited. Given that they can register as both a DH and DT, studies including these professions were also included. We defined each of these roles using descriptions by Satur [42]. Although the scope of practice of these roles can vary between countries and states, they generally have a similar scope of practice internationally [53].

### 2.3 Concept

After an initial search of the literature, the search was expanded to include other health contexts due to the limited evidence available with respect to multidisciplinary collaboration within RACFs including a DH, DT or OHT.

### 2.4 Context

As detailed above, other relevant contexts such as hospitals, primary care settings and university health centres were considered in relation to multidisciplinary collaboration.

### 2.5 Types of Sources

To capture a wide breadth of evidence, no limits were placed on the type of evidence. Thus, no specific outcomes of interest were defined. In relation to the second research question, interventions were considered if at least one outcome measure was an indicator of oral health, or a general health outcome that was acted upon through oral health care.

### 2.6 Search Strategy

A search strategy was developed to identify relevant studies according to the JBI method [45]. The full search strategy is presented in Appendix 1.

The search was undertaken between 1st May 2022 and 31st July 2022. The search strategy for peer‐reviewed articles had three steps. Firstly, a preliminary search of PubMed and CINAHL was conducted to find additional keywords to add to the search strategy (none identified). Secondly, the search strategy was applied to PubMed, CINAHL, Cochrane Library, PsychInfo, Scopus and Web of Science. Thirdly, the reference lists of all retrieved articles were searched for additional studies.

The search strategy was further adapted to search for grey literature according to guidelines by Godin et al. [54] The grey literature databases Grey Matters, OpenGrey, The Grey Literature Report and WorldCat were searched with the same search terms used in the peer‐reviewed articles strategy. A Google search was conducted to search for online non‐academic papers, and the first five pages of results were screened for relevance. Search terms are presented in Appendix 1. The websites of relevant organisations were manually searched for relevant papers. Organisations included national agencies for health information (e.g., Australian Institute of Health and Welfare) and national dental agencies (e.g., Australian Dental Association).

### 2.7 Study Selection

All identified studies were downloaded to a reference management software [55] and consolidated to remove duplicates. The screening process was facilitated by a software tool designed for screening abstracts for systematic reviews [56].

The titles, abstracts and/or executive summaries were screened against the inclusion criteria. For some studies, the full text was searched for keywords if the abstract contained insufficient information. All potentially relevant studies were then retrieved in full and assessed against the inclusion criteria in detail independently by authors KW and LS. Any disagreements were discussed by the researchers to ensure consensus. The PRISMA‐ScR Checklist is presented in Appendix 2 to ensure methodological transparency [57].

Data were charted using a modified version of the JBI source of evidence template [51] (see Appendix 3). Given the heterogeneity of the included studies, the charting process was iterative, in order to enable a flexible and study‐specific extraction process. Data were charted to summarise the nature of the evidence and to address the review questions. Information related to the feasibility of integrating DHs into RACFs was charted based on the framework of Bowen et al. [58] This framework defines areas of focus for feasibility studies and possible outcomes of interest relating to these areas. These areas are acceptability, demand, implementation, practicality, adaption, integration, expansion and limited‐efficacy testing.

### 2.8 Presentation of Results

The review findings are presented in tabular and narrative forms.

## 3 Results

**FIGURE 1:**
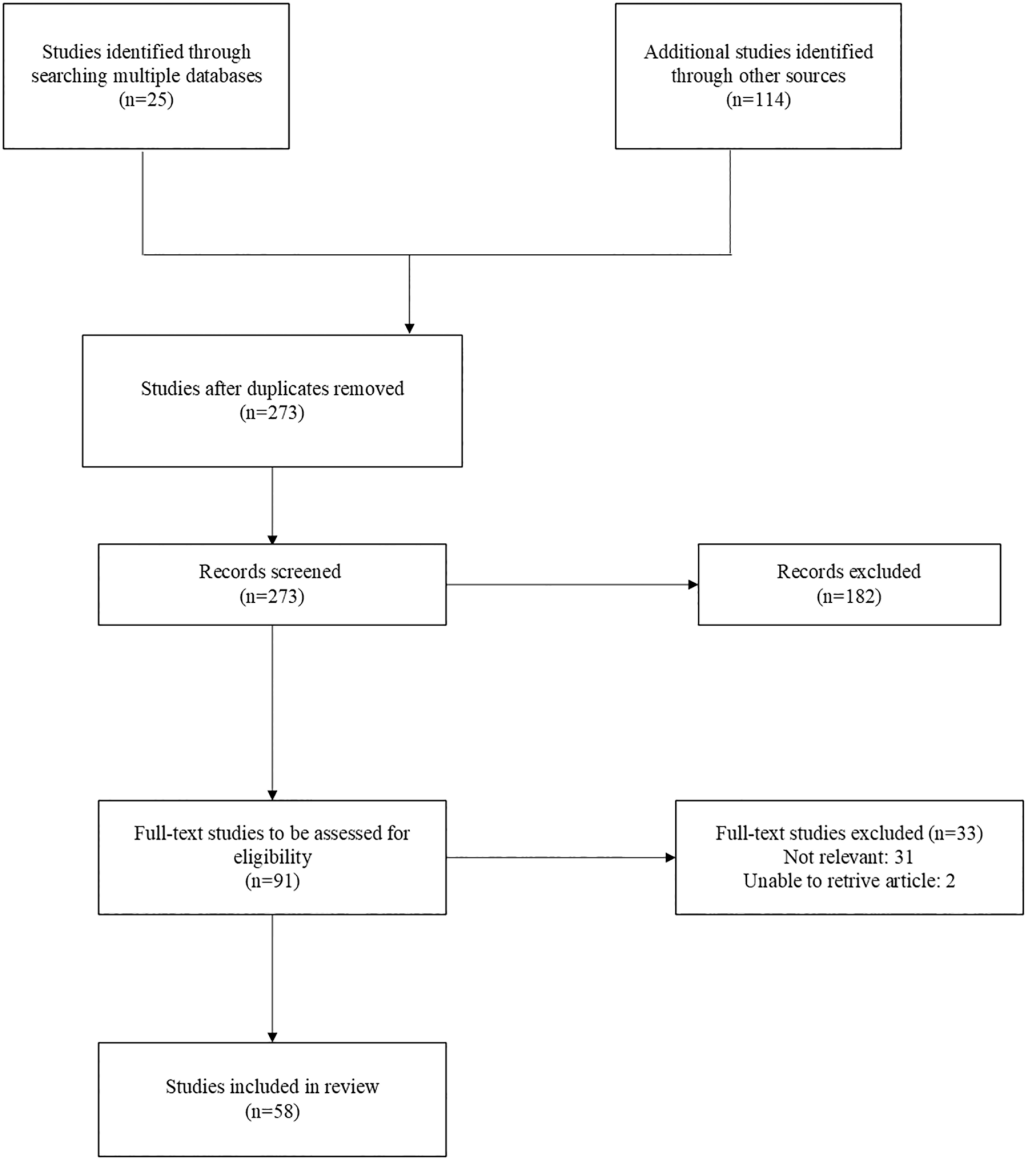
Search results and source selection and inclusion process [50].

The search returned 364 results. After removing duplicates, 273 records remained, and the titles and abstracts were screened for potentially relevant studies. Ninety‐one studies were identified as potentially relevant and were assessed against the inclusion criteria. The review finally included 58 studies (see Figure 1).

### 3.1 Characteristics of Included Studies

Fifteen studies (26%) were quantitative. Seven studies (12%) were randomised clinical trials and six (10%) were pre‐post studies. One controlled clinical trial (2%) and one comparative study (2%) were also included. Forty‐three (74%) of the included studies were qualitative. Ten were interviews (17%), nine were literature reviews (16%) and nine were case studies (16%). Five surveys and questionnaires (9%), four focus groups (7%), two workshops (3%), two descriptive phenomenological studies (3%), two review articles (2%), one systematic review (2%) and one review article (2%) were also included.

Forty studies were within the context of RACFs (70%). The 18 studies (31%) that were not within the RACF context all pertained to multidisciplinary collaboration. All but three studies (95%) included DHs as the primary dental professional. The other three included an OHT. (table summarising included studies available in Tables S1 and S2).

### 3.2 Review Findings

#### 3.2.1 Review Question 1: Is the Integration of DHs Into RACFs Feasible? If So, How Might This Look?

Of the 58 included studies, none evaluated the feasibility of integrating DHs into RACFs using a formal framework or objective measures. Feasibility of integration initiatives was mentioned using synonymous phrases such as ‘… shows potential to improve oral health outcomes’, [41] ‘…are capable’ [37], ‘… may positively impact oral health’, [59] ‘… is successful and transportable’ [40] and ‘…possible to implement’. [60]

Twenty‐three studies [37, 38, 40, 41, 59, 60, 61, 62, 63, 64, 65, 66, 67, 68, 69, 70, 71, 72, 73, 74, 75, 76, 77, 78] indirectly discussed at least one aspect of feasibility defined by Bowen et al. [58] (table available in Tables S1 and S2). Information regarding feasibility was gained through participant responses, feedback from the professionals implementing the program or literature reviews. The identified strategies were categorised as either support‐focused or service‐focused strategies.

### 3.3 Support‐Focused Strategies

Support‐focused strategies were defined as any strategy where the DH did not directly provide oral health services, but rather supported RACF staff to administer oral health care to residents. Nine studies utilised at least one support‐focused strategy [40, 41, 59, 62, 63, 71, 72, 73, 75]. An overview of these strategies is provided in Table 1.

**TABLE 1:** Summary of support‐focused integration strategies.

Strategy	No. papers per strategy	No. quantitative studies	No. qualitative studies
Theoretical education	5	4	1
Hands‐on training	5	5	0
Personalised care plan	3	3	0
Oral health champion	3	3	0
Ongoing support	7	2	0

The reporting on feasibility measures was mixed. Wintch et al. reported that support‐focused strategies were favoured by RACF executive directors compared to hiring dental professionals as staff, as it may be a more ‘economically sound business model’ [64]. Volk et al. [63] also reported that their program ‘involved relatively modest resource outlay’. Kullberg et al. [72] reported that RACF staff believed they had gained more knowledge about oral care. Conversely, Seleskog et al. [73] reported that educating RACF staff resulted in the opposite of the intended effect; participants became less confident in their ability to provide oral care. The authors theorised that this was due to the staff acquiring more insight into the difficulties of providing oral care. Volk et al. [63] directly assessed perceived program success and sustainability at a 12‐month follow‐up. RACF staff and DHs rated the perceived consistency in implementation out of 10 (10 = highly consistent). Scores ranged from 2.4 to 6.4, with the overall success of implementation rated 5.1.

Identified barriers to implementing this strategy include RACF staff lacking time to provide oral care [63, 73, 76] or to attend education/training sessions [72, 75]; high RACF staff turnover rates [64, 65]; RACF staff lacking confidence to provide oral care, particularly to resistant and high‐care residents [59, 73]; and resistance from RACF staff [60]. The barriers to successful implementation—particularly high staff turnover—are not easily overcome and thus may limit the strategy's success in improving oral health outcomes.

### 3.4 Service‐Focused Strategies

Service‐focused strategies were defined as any strategy where the DH provided a direct service to RACF residents. Thirteen studies utilised at least one service‐focused strategy. Four studies used a service‐focused strategy in combination with at least one support‐focused strategy [37, 38, 55, 60]. Table 2 provides an overview of these strategies.

**TABLE 2:** Summary of service‐focused integration strategies.

Strategy	No. papers per strategy	No. quantitative studies	No. qualitative studies
Professional oral health care	8	5	2
Examinations	4	3	1
Referral pathway	3	3	0
Prescription of devices/products	2	2	0

Reporting on feasibility outcomes was generally positive. Hopcraft et al. found that there ‘was excellent agreement between the dentist and DHs regarding the decision to refer residents to a dentist for treatment’ and that the DHs ‘are capable of formulating appropriate dental hygiene treatment plans’. [37] The DHs in this study had no prior experience of working in RACFs or with functionally dependent older adults. Six studies interviewing RACF staff demonstrated that there is high demand for DHs to provide oral health services, particularly on‐site [48, 64, 69, 70, 76, 77]. Tynan et al. [41] used a combination of on‐site visits and tele‐dentistry. RACF staff reported satisfaction with the program, and that it was appropriate for the RACF setting. Personalised plans were particularly useful for residents with dementia, as they could use oral care methods that minimised distress.

Two out of the three studies that reported a negative or mixed feasibility measure were student placement programs [61, 78]. Students reported unwelcoming attitudes from RACF staff, not feeling part of the healthcare team, not being able to communicate and interact with RACF staff and residents effectively [61], feeling inadequately prepared and not feeling confident in their role until half‐way through the placement [78]. Despite this, the students in both studies reported an appreciation of the placement at its conclusion.

Wallace et al. reported that the DH initially found assimilation into the daily functions of the RACF difficult, and staff and residents were often resistant to oral health practices [40]. Despite this, the program has since been adopted in other RACFs, suggesting that the program overall was feasible. Wintch et al. [64] reported that the higher cost of employing a dental professional as a full‐time staff member (rather than an educator) may prevent their integration as service providers in RACFs.

Barriers to successful implementation of a service‐focused strategy included lack of on‐site equipment [67]; DHs' lacking skills to work with patients with dementia and high‐care needs [40, 41, 61]; resistance from residents and staff towards oral health practices [61, 78]; difficulty assimilating into the daily functions of the RACF [78]; and not enough time to manage the program [41].

### 3.5 Review Question 2: What Strategies Can Be Used to Promote Effective Multidisciplinary Collaboration Between DHs and Other Health Professionals in RACFs?

While many studies recommended or utilised DHs and other health professionals working together in a RACF, no studies involving a DH in a multidisciplinary team within a RACF were identified. Seven included studies discussed collaboration within the context of RACFs [38, 41, 61, 66, 74, 79, 80]; however, collaboration was not the main focus of all seven studies. Strategies for promoting collaboration were consequently also derived from additional studies [79, 80, 81, 82, 83, 84, 85, 86, 87, 88, 89, 90, 91, 92, 93, 94, 95, 96, 97, 98] discussing multidisciplinary collaboration in different healthcare settings. Table 3 summarises the findings on facilitating multidisciplinary collaboration.

**TABLE 3:** Summary of findings related to multidisciplinary collaboration.

First Author (Year)	Findings
Atchison (2018)	Dedicated personnel and electronic tools should be used by all team members to facilitate referrals and care coordination, scheduling of appointments and multidisciplinary consultations Appointed case managers can identify residents who need follow‐up or preventive services and to convert episodic or emergency dental service users to routine care
Bisset (2020)	Signposting a patient with suspected health issues to a staff member/health professional for investigation helped facilitate collaboration
Blue (2016)	Dental and medical records must be integrated to support streamlined and comprehensive care
Bowes (2010)	Effective collaboration required an environment of trust and recognition of each members' expertise
Braun (2021)	A supportive team leader and streamlined workflow, particularly for scheduling and billing helped successful collaboration It is important that team members are adaptable and problem solvers, have a willingness to “learn‐by‐doing” and are willing to work with challenging populations
Braun (2016)	Having a DH on‐site facilitated multidisciplinary collaboration by making them a visible/present member of the healthcare team The dental professional should be comfortable working in non‐traditional settings Shared triage and care plans, medical and dental records that share patient information and commonly supported scheduling and billing facilitated multidisciplinary collaboration
Coleman (2005)	Education programs may facilitate multidisciplinary training and collaboration Appointing a representative nurse onto relevant dental boards and vice versa to deal with multidisciplinary relations may facilitate collaboration Initiating a multidisciplinary project around oral health can facilitate multidisciplinary collaboration
Coleman (2006)	Educational experiences should be coordinated among dental and nursing schools to promote multidisciplinary collaboration and provide greater geriatric experience DHs could be hired as RACF staff members to provide routine care and administer care as established in the care plans
Compton (2013)	Increasing awareness in RACF staff regarding the roles of dental professionals/students may lead to more communication and interaction Protocols should be developed to ensure referrals, and recommendations for residents are follow‐up with by RACF staff Scheduling of professional/student contact hours should coincide with the reschedules of the RACF staff
Duley (2012)	Students should be provided with collaborative experience within their education so that they are better prepared to work in a multidisciplinary team
Grant (2017)	A formal structure of operation through concrete strategies and protocols are necessary so each members knows their role as well as the role of others Being present and willing to help each other, trust and value in each other and strong leadership can motivate team members towards high performance
Hachey (2020)	Oral health education should be incorporated into non‐oral health academic programs to facilitate understanding of the relationship between oral and general health
Huynh (2017)	Interdisciplinary care conferences can provide a platform for multidisciplinary discussion so that a collaborative approach to care that included oral health can be developed
Luebbers (2021)	The organisations system should allow for easy transfer of data between healthcare professionals
MacEntee (2011)	Team members need practical exposure to the cultures of other team members to ensure the team's effectiveness (e.g., use of acronyms in different professions hinder effective collaboration if not understood by all)
Nakajima (2021)	Team members should have opportunities to discuss cases and patients with other team members and to discuss disagreements or issues and resolve them.
Niesten (2021)	Clear guidelines and protocols are required to streamline the workflow and referral pathways between team members Interprofessional training, both in formal education and on‐the‐job is beneficial Opportunities for professionals to teach other and share knowledge, particularly regarding challenging populations, was seen as beneficial by health professionals A central information system for storage and exchange of patient data is necessary
Persson (2016)	The responsibilities of each team member must be made clear, particularly regarding administrative work An on‐site presence can make oral health issues more visible and help more available to others Dialogue and opportunities where knowledge can be shared, and professional networks can be established are important Establishing a common goal which is collaboratively addressed can facilitate collaboration
Simon (2019)	Dental professionals should have access to health records so that all treatment is recorded and accessible to the entire multidisciplinary team. Notes from dental professionals should be accessible from the same interface as all other medical notes Other members of multidisciplinary teams should be educated about the role of the dental professional through multiple avenues (e.g., emails, fliers and in‐person) Educating staff on the importance and safety of preventive oral services may increase the willingness to collaborate with dental professionals
Swanson Jaecks (2009)	Important for team members to have good communication and leadership skills to work effectively with others
Theile (2016)	Good management skills, communication strategies and leadership qualities are required to coordinate the interprofessional provision of comprehensive healthcare The role of each team member should be clear

Six studies highlighted the importance of sharing of patient records through centralised, electronic systems [74, 81, 82, 84, 89, 94]. This would create a more streamlined workflow and efficient communication in terms of scheduling as well as sharing of important health information. For example, it would be beneficial for DHs to check patient records to see whether a patient is prescribed blood thinning medications, which is common in older adults [99], due to risk of bleeding during dental procedures. This may be useful for residents with dementia who cannot communicate or remember their medications.

Seven studies recommended clearly defined roles and responsibilities of each team member as a facilitator to collaboration [61, 69, 74, 89, 91, 93, 95]. The responsibility for administrative tasks such as scheduling and billing [61, 81, 84, 85, 89] should be clarified to further ensure a streamlined workflow by avoiding issues such as scheduling conflicts. This should also be centralised, as fragmentation of services was identified as a barrier to successful collaboration [85, 93]. One strategy could be to appoint an administrative staff as the oral health ‘champion’ who can coordinate oral health services and liaise with dental professionals and other health care team members [41, 81]. It is also important to educate team members on the role of the dental professional, given that low oral health literacy of non‐dental team members can hinder collaborative efforts [69, 89], most RACF staff lack oral health knowledge [14], and educating team members on the importance of oral health and the safety of preventive dental treatments may increase willingness to work alongside dental professionals [89]. This should be a reciprocal learning experience, so that the dental professionals can learn about other disciplines [90, 96].

### 3.6 Review Question 3: Can the Integration of DHs Into RACFs Improve Oral Health and Other Health Outcomes of Residents With Dementia?

Table 4 summarises the findings on improving oral health in residents with dementia. No studies focused exclusively on residents with dementia. Participants included both cognitively normal and cognitively impaired residents. Two studies utilised support‐focused strategies alone [59, 72], four utilised service focus strategies alone [68, 100, 101, 102], and five studies utilised both [40, 62, 63, 73, 103]. Additionally, a review by Siegel et al. [104] was considered. The authors investigated the effectiveness of a range of oral health interventions involving or led by a DH. The summary of their findings is included in Table 4.

**TABLE 4:** Summary of findings related to improving oral and general health outcomes in RACFs.

First Author (Year)	Outcome measures	Findings
Adachi (2007)	*Oral Health Indicators:* Dental Plaque Index Tongue Plaque Index *Influenza:* Rapid Antigen Detection Test (QuickVue Kit) *Pathogens:* Cultivable bacterial cell numbers (*Staphylococcus*) Neuraminidase Assay Kit *Fever:* Temperature ≥ 37.8°C	Preventive oral health care by DHs was effective in preventing influenza (*p* = 0.008), fatal aspiration pneumonia (*p* < 0.05), fevers (*p* < 0.05) and reducing the prevalence of respiratory pathogens in RACF residents, including those with dementia
Amerine (2014)	Oral Health Assessment Tool	The facilities that received the intervention showed significant improvements on the Oral Health Assessment Tool in at least one area (Facility A = tongue health, denture status, oral cleanliness, Facility B = tongue health). The control (Facility C) showed no improvements
Ishikawa (2008)	*Oral Health Indicators:* Mean no. of teeth present Mean no. of decayed teeth Decayed, Missing and Filled Permanent Teeth (DMFT) index % Edentulous % Denture wearers Debris Index Pocket Depth *Pathogens:* Mean no. (*Streptococci, Staphylococci, Candida, Pseudomonas*, Black‐pigmented *Bacteroides* species) *Febrile Days* *Aspiration pneumonia*	Oropharyngeal bacteria decreased or disappeared in all three facilities postintervention Scores on the debris index significantly decreased in Facility A (which received the most professional oral care). There was no difference in Facility B or C
Kullberg (2010)	*Gingival Bleeding Index* *Plaque Index*	A statistically significant reduction in gingival bleed scores and plaque scores were observed postintervention (*p* < 0.001) Post hoc analysis revealed that the increased use of electric toothbrushes postintervention did not contribute statistically significantly to the reduction in gingival bleeding scores or plaque scores
Marchini (2018)	*Oral Health Indicators:* Dental Plaque Index Denture Plaque Index Self‐reported dry mouth No. oral lesions No. teeth Bleeding on brushing DMFS and DMFT Indices *Febrile Days* *Pneumonia:* X‐ray *Pathogens:* Total CFU Count (*Porphyromonas gingivalis, Fusobacterium nucleatum, Actinomyces viscosus, A* *. actinomycetemcomitans* , *Candida albicans*)	There were no statistically significant differences among the intervention groups for any of the recorded clinical or microbiological outcomes
Morino (2014)	Denture Plaque Index Plaque Index for Long‐Term Care Gingival Index for Long‐Term Care Minimum Data Set (presence or absence of inflamed or bleeding gums item only)	A statistically significant improvement in Dental Plaque Index scores was observed in the intervention group. In the intervention group, the Dental Plaque Index scores of functionally dependent residents significantly decreased (*p* < 0.05), but no statistically significant change was observed in residents who were functionally independent
Seleskog (2018)	Revised Oral Assessment Guide Dental Plaque Index Gingival Index	Two items on the Revised Oral Assessment Guide showed improvement in the intervention group (lips and gums). A statistically significant decrease in dental plaque was observed in the intervention group
Siegel (2017)	N/A	Results on the effectiveness of preventive oral health care by a DH were mixed. Five studies found statistically significant improvements in indicators of oral health. Two studies found no improvement in plaque or respiratory infection rates
Sloane (2013)	Denture Plaque Index	Scores on plaque (*p* < 0.001), gingival (*p* < 0.001) and denture plaque (*p* < 0.04) indices significantly improved postintervention
Volk (2020)	Simplified Oral Hygiene Index Denture Plaque Index Plaque Index for Long‐Term Care Gingival Index for Long‐Term Care	73% of RACFs reported improvements in plaque, gingiva or denture plaque scores at either 6‐month or 12‐month follow‐ups 41% reported improvements in all three at both 6‐month and 12‐month follow‐ups
Wallace (2016)	Silness and Loe Plaque Index	The intervention by the DH statistically significantly reduced plaque scores in RACF residents (*p* < 0.001) Pneumonia, febrile days and death from pneumonia significantly decreased in the intervention group
Yoneyama (2002)	Debris Index *Pneumonia:* Chest radiograph One symptom (cough, temperature > 37.8°C, or subjective dyspnoea) *Febrile days:* Axillary temperate > 37.8°C	Oral care significantly reduced the Debris Index in contrast to non‐oral care group (*p* < 0.01) The mortality due to pneumonia in the intervention group was about half of that in the control group

Abbreviations: CFU, Colony‐Forming Unit; DH, dental hygienist; DMFS, Decayed, Missing and Filled Surfaces; DMFT, Decayed, Missing and Filled Permanent Teeth; RACF, residential aged care facility.

All but one study reported a statistically significant improvement in at least one measured outcome [103]. Marchini et al. stated that low response rates and high attrition rates led to the study being underpowered [103]. The findings of these studies indicate that integrating a DH into RACFs using both support‐focused and service‐focused strategies may have a positive impact on the oral health of RACF residents with dementia.

## 4 Discussion

This scoping review aimed to map the current evidence on the integration of DHs in RACFs and their multidisciplinary teams, with a focus on improving the oral health of residents with dementia. Two types of integration strategies emerged, and various strategies for multidisciplinary collaboration were identified. However, the current evidence supporting their feasibility is limited. Despite this, both strategies show promise in improving the oral health of RACF residents with dementia.

A strength of this study is that a systematic and iterative approach to identify relevant papers was used. However, despite efforts to capture a breadth of literature, it is possible that relevant papers were not captured due to time constraints placed on the search process and data extraction only being performed by one reviewer. It is also possible that relevant papers were not identified due to the keywords in the paper not matching the terms in the search strategy. This may especially be the case for papers where the main focus was not on the concepts used to create the search strategy. It was determined that no additional studies were published in the time between the study selection and submission that would meet inclusion criteria or meaningfully change the reported results.

This study used definitions provided by Satur [42] to define the roles of DHs, OHTs and DTs. It is important to note that the scope of practice for dental professionals varies by country and even by state. This may limit the generalisability of our findings beyond the scope of Australia. However, the health promotion role of DHs is less variable globally [53] and the ability to perform more complex treatments that vary among countries would likely be limited in RACFs due to limited dental equipment in RACFs.

### 4.1 Research Gaps

Among the included studies, there was little reporting on the feasibility of integration strategies in practice. Assessing feasibility is important, as it enables researchers and policy makers to evaluate whether research findings are relevant and sustainable in practice [58]. Future studies investigating the role of DHs in RACFs should endeavour to directly evaluate the feasibility integration using formal evaluation frameworks.

There were a variety of study designs and methodologies in the included studies. To further evaluate the effectiveness of integration strategies and multidisciplinary teams in improving oral health in RACFs, more experimental research is required. However, there are many barriers to conducting robust experimental research such as RCTs in RACFs. This includes issues inherent to RACFs [105], such as administrative and logistical barriers, issues inherent to research in older populations (high attrition and obtaining informed consent), as well as the low prioritisation of oral health in RACFs [14]. Barriers to conducting experimental research can be reduced by addressing them in the planning phase of research and should be discussed with stakeholders [105].

There were few examples of multidisciplinary collaboration within RACFs with health professionals other than nurses. Nevertheless, the inclusion of setting other than RACFs to identify facilitators of multidisciplinary collaboration provided insight to how dental professionals can function within multidisciplinary healthcare teams. While it is important for dental professionals and nurses to have a close working relationship within RACFs, further research is needed to determine the effectiveness of dental professionals in a team with a wide range of professionals. This should include allied health professionals such as psychologists and speech language pathologists that commonly work with dementia patients.

There is also a need for studies specifically focused on residents with dementia, given the difficulty and reluctance towards providing oral health care for this population [10, 11, 12, 13, 14, 69]. This is important to ensure that the findings of the included studies do not diminish when only residents with dementia are considered. More consistent and stringent reporting on the number of participants with dementia and the level of functional impairment could provide further evidence that an intervention strategy will be feasible for residents with varying degrees of functional impairment. This could be achieved by including a measure of functional dependence, such as the Katz Index of Independence in Activities of Daily Living [106].

## 5 Summary and Conclusion

This scoping review summarised the current evidence supporting the integration of DHs into RACFs, with a focus on providing care to residents with dementia and working in a multidisciplinary team. Strategies to integration were categorised as support‐focused and service‐focused. More evidence is needed to confirm their feasibility and effectiveness in improving oral health status, particularly RCTs and other experimental studies. Presently, DHs are underutilised in multidisciplinary teams, reflecting the low prioritisation of oral health in RACFs. Examples from collaboration in other health settings presented in this study can guide decision‐making in integrating DHs into RACF multidisciplinary teams.

## Author Contributions

**Kelsey West:** conceptualisation, methodology, investigation, writing – original draft and visualisation. **Julie Saunders:** conceptualisation, methodology, writing – review and editing and supervision. **Linda Slack‐Smith:** conceptualisation, methodology, writing – review and editing and supervision.

## Ethics Statement

The authors have nothing to report.

## Conflicts of Interest

The authors declare no conflicts of interest.

## Supporting information


Table S1.

Table S2.

Appendix S1.

## Data Availability

As this is a review, there is no new data generated.

## Appendix 1 Search Strategy

**Table:**

Search line	Index term combinations
**Search Strategy for Peer‐Reviewed Articles**	
S1	(“oral health therap*” OR “dental therap*” OR “dental hygienist” OR integrated OR multidisciplinary OR “healthcare team” OR “primary care team”) AND (“aged care” OR “nursing home” OR “long‐term care facility” OR “assisted‐living facility”) AND (dementia OR “Alzheimers disease”)
S2	(“oral health”) AND (intervention OR program*) AND S1
S3	(barriers OR facilitators) AND (“oral health therap*” OR “dental therap*” OR “dental hygienist” OR integrated OR multidisciplinary OR “healthcare team” OR “primary care team”) AND (“aged care” OR “nursing home” OR “long‐term care facility” OR “assisted‐living facility”)
S4	(“oral health therapist” OR “dental hygienist” or “dental therapist”) AND (“multidisciplinary” OR “interdisciplinary” OR “transdisciplinary” OR “collaborative” OR “multiprofessional” OR “interprofessional”) AND (strategies OR implement OR facilitate OR integrate OR “health care team” OR “primary team”)
**Search Strategy for Grey Literature**	
S1	Oral health therapist AND dementia OR aged care
S2	Dental therapist AND dementia OR aged care
S3	Dental hygienist AND dementia OR aged care
S4	Oral health promotion AND aged care OR dementia
S5	Oral health AND dementia AND guidelines OR policies
S6	Oral health AND dementia AND program OR initiative OR intervention
S7	Multidisciplinary AND oral health therapist OR dental hygienist OR dental therapist

## Appendix 2 PRISMA‐ScR Checklist

Preferred Reporting Items for Systematic reviews and Meta‐Analyses extension for Scoping Reviews (PRISMA‐ScR) Checklist.

**Table:**

Section	Item	Prisma‐ScR checklist item	Reported on page no.
**Title**			
Title	1	Identify the report as a scoping review	p. 1
**Abstract**			
Structured summary	2	Provide a structured summary that includes (as applicable) background, objectives, eligibility criteria, sources of evidence, charting methods, results and conclusions that relate to the review questions and objectives	p. 3
**Introduction**			
Rationale	3	Describe the rationale for the review in the context of what is already known. Explain why the review questions/objectives lend themselves to a scoping review approach	p. 4–5
Objectives	4	Provide an explicit statement of the questions and objectives being addressed with reference to their key elements (e.g., population or participants, concepts and context) or other relevant key elements used to conceptualise the review questions and/or objectives	p. 6
**Methods**			
Protocol and registration	5	Indicate whether a review protocol exists; state if and where it can be accessed (e.g., a web address); and if available, provide registration information, including the registration number	p. 7
Eligibility criteria	6	Specify characteristics of the sources of evidence used as eligibility criteria (e.g., years considered, language and publication status) and provide a rationale	p. 7
Information sources^a^	7	Describe all information sources in the search (e.g., databases with dates of coverage and contact with authors to identify additional sources), as well as the date the most recent search was executed	p. 8
Search	8	Present the full electronic search strategy for at least 1 database, including any limits used, such that it could be repeated	Appendix 1
Selection of sources of evidence^b^	9	State the process for selecting sources of evidence (i.e., screening and eligibility) included in the scoping review	p. 9
Data charting process^c^	10	Describe the methods of charting data from the included sources of evidence (e.g., calibrated forms or forms that have been tested by the team before their use, and whether data charting was done independently or in duplicate) and any processes for obtaining and confirming data from investigators	p. 9–10
Data items	11	List and define all variables for which data were sought and any assumptions and simplifications made	Appendix 1
Critical appraisal of individual sources of evidence^d^	12	If done, provide a rationale for conducting a critical appraisal of included sources of evidence; describe the methods used and how this information was used in any data synthesis (if appropriate)	N/A
Synthesis of results	13	Describe the methods of handling and summarising the data that were charted	p. 9–10
**Results**			
Selection of sources of evidence	14	Give numbers of sources of evidence screened, assessed for eligibility and included in the review, with reasons for exclusions at each stage, ideally using a flow diagram	p. 10
Characteristics of sources of evidence	15	For each source of evidence, present characteristics for which data were charted and provide the citations	p. 10
Critical appraisal within sources of evidence	16	If done, present data on critical appraisal of included sources of evidence (see item 12)	N/A
Results of individual sources of evidence	17	For each included source of evidence, present the relevant data that were charted that relate to the review questions and objectives	Table S1
Synthesis of results	18	Summarise and/or present the charting results as they relate to the review questions and objectives	p. 11–16
**Discussion**			
Summary of evidence	19	Summarise the main results (including an overview of concepts, themes and types of evidence available), link to the review questions and objectives and consider the relevance to key groups	p. 17
Limitations	20	Discuss the limitations of the scoping review process	p. 17
Conclusions	21	Provide a general interpretation of the results with respect to the review questions and objectives, as well as potential implications and/or next steps	p. 19
**Funding**			
Funding	22	Describe sources of funding for the included sources of evidence, as well as sources of funding for the scoping review. Describe the role of the funders of the scoping review	p. 2

Abbreviations: JBI, Joanna Briggs Institute; PRISMA‐ScR, Preferred Reporting Items for Systematic reviews and Meta‐Analyses extension for Scoping Reviews. *Source:* Tricco et al. [57].

^a^ Where *sources of evidence* (see the second footnote) are compiled from, such as bibliographic databases, social media platforms and web sites.

^b^ A more inclusive/heterogeneous term used to account for the different types of evidence or data sources (e.g., quantitative and/or qualitative research, expert opinion and policy documents) that may be eligible in a scoping review as opposed to only studies. This is not to be confused with *information sources* (see the first footnote).

^c^ The frameworks by Arksey and OMalley [6] and Levac and colleagues [7] and the JBI guidance [4, 5] refer to the process of data extraction in a scoping review as data charting.

^d^ The process of systematically examining research evidence to assess its validity, results and relevance before using it to inform a decision. This term is used for items 12 and 19 instead of ‘risk of bias’ (which is more applicable to systematic reviews of interventions) to include and acknowledge the various sources of evidence that may be used in a scoping review (e.g., quantitative and/or qualitative research, expert opinion and policy document).

## Appendix 3 Modified JBI Source of Evidence Template

**Table:**

Scoping review details	
Scoping review title	Integrating dental professionals into Australian aged care: A scoping review
Review objectives	1. Review the current literature and identify any gaps in the evidence base 2. Summarise and disseminate key research findings in a manner that is easily accessible and understandable to relevant stakeholders 3. Provide preliminary recommendations for how to integrate OHTs into the RACF system
Review questions	1. Is the integration of DHs into RACFs feasible? If so, how might this look? 2. What strategies can be used to promote effective multiple disciplinary collaboration between DHs and other health professionals in RACFs? 3. Can the integration of DHs into RACFs improve the oral health of residents with dementia?
**Inclusion/Exclusion Criteria**	
Population *(Older adults living in a RACF, who have dementia **OR** OHTs, DHs or DTs)*	
Concept *(The provision of dental care to older adults with dementia, within RACFs by an OHT, DH or DT **OR** Strategies for integrating OHTs, DHs or DT into RACFs **OR** Strategies for integrating OHTs, DHs or DTs into a multidisciplinary healthcare team)*	
Context *(RACFs **OR** setting where an OHT, DH or DT is part of a multidisciplinary healthcare team)*	
Types of evidence source *(all types)*	
**Evidence source details and characteristics**	
Citation details *(author/s, date and title)*	
Country	
Aims/objectives	
Relevant professionals involved	
Participants details (*age, sex, number and cognitive/functional status*)	
**Details/results extracted from source of evidence**	
Improvements in oral health status *(include outcome measures)*	
Tasks/duties/role of OHT/DH/DT within the RACF	
Strategies for multidisciplinary collaboration	
