# Supplementary material for: Integrating Dental Professionals Into Aged Care With Focus on Australia: A Scoping Review
Source: Gerodontology. 2025 Jan 12;42(2):147–64. doi: 10.1111/ger.12784 (PMC12106945; doi:10.1111/ger.12784)
Supplement: Supplementary file 1 — Table S1. [file GER-42-147-s001.docx]

**Summary of Included Studies**

| First Author (Year)  Country; Setting | Study Methodology (groups); length  Total n; % with dementia | Purpose | Professional(s) Involved | Study Description |
| --- | --- | --- | --- | --- |
| Aagaard (2020); Denmark; RACF | Case study | To evaluate the process of implementing an oral care intervention in nursing homes in a Danish municipality. | DHs, dentists, RACF managers, RACF care professionals | An embedded multiple-case study combined with principles of realist evaluation utilised observations, six group interviews and 22 face-to-face interviews in three RACFs. |
| Adachi (2007);  Japan; RACF | Comparative study (2 groups); 12 months  n= not stated; Unspecified | Determine numbers of microorganisms, potent pathogens of respiratory infection, enzymatic activity in saliva, fevers, prevalence of fatal aspiration pneumonia and prevalence of influenza. | DHs | Intervention Group: Residents across 2 RACFs received professional teeth cleaning from a DH.  Control Group: Usual routines were followed |
| Amerine (2014); USA; RACF | Pre-post study (3 groups); 8 weeks n= 79; 100% with cognitive impairment | Examined impact of onsite  support by a dental hygiene champion  on oral health and quality of life  of elderly residents in three RACFs | DHs, RACF staff | Facility A (n=27): Received standardized oral health education/materials with onsite DH support. Education/materials included an educational session and an oral health protocol guidebook.  Facility B (n=31): Received standardized oral health education/materials only.  Facility C (n=20): Control site. |
| Atchison (2018)  USA; various | Environmental Scan/Case Study | Describe the status of health care integration of oral health and primary care services. | DHs | The authors chose four programs as case studies and interviewed key personnel in each program. One case study illustrates each category of integrated services; additional examples describe category variation. |
| Bisset (2020)  UK; primary care practices | Workshops  n=43 | To explore interprofessional communication and collaboration in guideline-concordant diabetes and periodontitis care. | DHs, dentist, practice managers, GPs | Six iterative workshops in two medical practices and two dental practices were conducted to identify ways to improve multidisciplinary working attended by staff from medical and dental primary care practices, and people with diabetes |
| First Author (Year)  Country; Setting | Study Methodology (groups); length  Total n; % with dementia | Purpose | Professional(s) Involved | Study Description |
| Blue (2016)  USA; primary care | Review Article | To describe the role of a DH within an Accountable Car Organisation framework within primary care | DHs | A dental insurance Chief Operating Officer and a dental hygiene educator used their unique perspectives and experience to describe the potential of an interdisciplinary team-based approach to individual and population health, including oral health, via an accountable care community. |
| Bowes (2010)  Canada; various | Case study | Illustrate a successful collaboration between nursing and dental hygiene and outline an ongoing collaboration. | DHs, nurses | The paper outlines an ongoing collaboration—an oral health community of practice that serves as an example of the synergy created when professionals work together towards a common goal. |
| Branch-Mays (2017)  USA; university dental clinic | Case study  n=190; n/a | Evaluate the feasibility of an interprofessional education and collaborative practice model and to report results of the needs assessment. | DHs, dentists, dental assistants, dental students, dental therapy students, pharmacy students, pharmacists | The team selected 190 patients in the clinic for the study based on the inclusion criteria: patients with two or more chronic medical conditions who were taking medications. They received a comprehensive dental exam, review of social and medical history, and medication therapy management assessment by the interprofessional team. |
| Braun (2021)  USA; medical practices | Interviews; n=17  Survey; n=390 | Explore the perceptions of DH and patient participants in the Colorado Medical Dental Integration project. | DHs | Interviews explored DH’s perceptions of working as an integrated DH, factors impacting implementation, the level-of-Integration into the medical team, and how ways to access to dental services were expanded through the project. A patient-participant survey assessed perceptions regarding the care. |
| Braun (2016)  USA; medical centres, community | Literature review | Explore four innovative care models aimed to expand access to dental care. | DHs | Current activities in Colorado and around the nation are described regarding the provision of basic preventive oral health services. |
| Brondani (2011)  Canada; RACF | Descriptive Phenomenology | To describe a geriatric community service-learning component, and to explore a project carried out by a group of first year dental students at a RACF. | Undergraduate dental students, DH, dentist | Three groups of first-year students were placed into a RACF for 19 half-days. They proposed and developed a project for health promotion within a RACF. The project was implemented and evaluated over 6 weeks. |
| First Author (Year)  Country; Setting | Study Methodology  (groups); length  Total n; % with dementia | Purpose | Professional(s) Involved | Study Description |
| Britton (2016)  Australia; RACF | Interviews  n=17; n/a | Investigate the perceptions of dentists, DH, and OHTs of barriers and enablers they face providing oral care to residents in RACFs. | OHTs, DHs, dentists | Semi-structured interviews were conducted to explore the experience of working in aged care or with older people, organisational factors that would support a dental professional working in aged care settings, dimensions of working in a team in these contexts, oral health promotion strategies, connections to public policy and understandings of the role of a health professional. |
| Coleman (2005)  USA; RACF | Literature review | Outline the need for greater attention to oral health care for the elderly in both nursing education and practice and describe opportunities for effective interprofessional collaboration between nursing and oral health professionals. | DHs, nurses | Provides are review of oral health care needs in older adults and provides specific recommendations for fostering multidisciplinary collaboration between DHs and nurses. |
| Coleman (2006)  USA; RACF | Literature review | Propose a model of care utilizing transdisciplinary collaboration between nursing and dental hygiene professionals to achieve a best practice approach to meeting oral health needs of RACF residents. | DHs, nurses | Provides a review of oral health trends in older adults, discusses barriers to oral care in RACFs and the impact on quality of life. A model of care is proposed. |
| Compton (2013)  USA; RACF | Focus groups  and interviews  n= 8; n/a | Report the qualitative data based on the first objective which was the socialization of DH students to the RACF. | DH students, DH instructors | Final year DH students participated in the ElderSMILES practicum within 2 RACFs for 2 days/week for 13 weeks. Two registered DH acted as clinical instructors and support students.  Focus groups (n=6): 2 groups, 3 DH students each  Interviews (n=2): DH instructors |
| Dahm (2015)  USA; RACF | Literature review | Identify systemic health conditions, oral health conditions, barriers to oral care for older adults and to offer recommendations for increased access to care within RACFs through the use of registered DHs. | DHs | The authors recommend 14 areas in which DHs could provide increased access to care for RACFs. |

| First Author (Year)  Country; Setting | Study Methodology (groups); length  Total n; % with dementia | Purpose | Professional(s) Involved | Study Description |
| --- | --- | --- | --- | --- |
| Duley (2012)  USA; university | Case study | Describe a Centre for Oral Health Promotion that would expand services offered in dental hygiene educational settings as well as expand dental hygiene, nursing, and health care management student experiences. | DH students, nursing students | The authors describe an education collaboration between dental hygiene and nursing students that provides collaborative experiences and to further understand the relationship between oral health and systemic health issues. |
| Hachey (2020)  Canada; Primary care | Case study | Describe a framework to guide oral health policy and practice that highlights the importance of collaboration between dental and other primary care providers. |  | The authors describe The Health Impact Pyramid, a paradigm to guide health policy and programming with ready application to oral health care in Canada for the delivery of evidence-based oral health interventions with high impact. |
| Hearn (2016)  Australia; RACF | Interviews  n=30; n/a | Explore RACF staff perspectives on how to engage dental professionals in the provision of oral care for RACF residents. Investigate the views of RACF staff on how to engage dental professionals. | Directors of nursing, clinical nurses, personal care assistants | Interviews were conducted with 6 directors of nursing, 14 clinical nurses, and 10 personal care assistants. The interviewers used open-ended questions. |
| Hopcraft (2011)  Canada; RACF | Comparative study;13 months  n=510; 38% with dementia | Determine whether a DH could undertake dental examinations for RACF residents, devise adequate periodontal and preventive treatment plans, and identify and refer patients who require treatment and assessment by a dentist. | DHs, dentists | Residents across 31 RACFs were examined by a dentist and one of 4 DHs, and their referral and treatment decisions were compared. None had undergone extra training in special needs or gerodontology, or had significant experience working in RACFs |
| Huynh (2017)  Canada; RACF | Interviews and focus groups (2 groups)  n=17; n/a | Explore how the participation of dental hygiene students in interdisciplinary care conferences in RACFs influenced the staff’s awareness of oral health, the student’s provision of care and the student’s ability to work in an interdisciplinary team. | DH students, RACF staff | Two focus groups were conducted (n=9 RACF; n=3 DH students). Five personal phone interviews were conducted with DH students. |

| First Author (Year)  Country; Setting | Study Methodology (groups); length  Total n; % with dementia | Purpose | Professional(s) Involved | Study Description | |
| --- | --- | --- | --- | --- | --- |
| Grant (2017)  Canada; rural communities | Literature review | Explore the outcomes of interprofessional relationships between dental hygienists and other health professionals on individuals residing in rural communities. | DHs | Themes addressed include: Implementing interprofessional education experiences in entry-to-practice health programs, interprofessional dynamics in primary health care teams, health perceptions in rural communities, and barriers and enablers to interprofessional relationships. |  |
| Ishikawa (2008)  Japan; RACF | RCT (3 groups); 5 months  n=202, 63.7% with cognitive impairment | Evaluate the longitudinal prevalence of oropharyngeal bacteria in RACF residents after professional mechanical cleaning of the oral cavity and/or disinfectant gargling, and to seek an effective method of administering oral health care. | DHs | Facility A (n=72; 54.2% cognitive impairment): Residents received professional oral health care once a week from a DH for 5 months.  Facility B (n=70; 67.1% cognitive impairment): Received professional care in last 3 months  Facility C (n=60; 70.0% cognitive impairment): Residents gargled with 0.35% providone iodine once a day after lunch for first 2 months. Then received professional care once a week in addition to gargling for 3 months. |  |
| Juhl (2016)  USA; hospitals | Case study | Describes a hospital-based educational experience for dental hygiene students and provides advocacy strategies for inclusion of dental hygienists within the hospital interdisciplinary team. | DH students, nurses, hospital staff (unspecified) | The authors collaboratively developed and implemented a hospital-based rotation within the curriculum of a dental hygiene educational program and used advocacy skills to encourage hospital administrators to include a dental hygiene presence within hospital-based care teams |  |
| Kullberg (2009)  Sweden; RACF | Pre-post study  (1 group); not stated  n=105; n/a | Describe a new dental hygiene education program for RACF staff and to report experiences from the program at a RACF. | DH, nursing staff, psychologist | Nursing staff members with a RACF dementia care department participated in a dental hygiene education program.  Step 1: Staff received hands-on training on tooth brushing techniques  Step 2: A DH and psychologist conducted small discussion groups, aimed at modifying negative attitudes and perceptions of oral hygiene tasks.  Step 3: A 90-minute theoretical lecture focusing on the association between oral health and general health. |  |

| First Author (Year)  Country; Setting | Study Methodology (groups); length  Total n; % with dementia | Purpose | Professional(s) Involved | Study Description |
| --- | --- | --- | --- | --- |
| Kullberg (2010)  Sweden; RACF | Pre-post study  (1 group);  3 weeks  n=43; 100%  with dementia | Evaluate the effect of a repeated dental hygiene education programme for nursing staff in a nursing home for older people. | DHs, nursing staff | Intervention group (n=43): nursing staff who received training 1.5 years earlier (see above) received additional education sessions from a DH. Staff received advice personalised for each resident and hands-on training.  All residents were provided an electric toothbrush and chlorhexidine gluconate 1% gel. |
| Lewis (2015)  Australia; RACF | Literature review | To review the Better Oral Health in Residential Care initiative, barriers to accessing oral health care for frail and functionally dependent older adults, and potential minimal intervention treatments. | OHTs, DHs, Dentists | The authors describe the Better Oral Health in Residential Care plan endorsed by the Australian Government in 2010. Minimal intervention treatments for frail and functionally dependent elderly were reviewed. |
| Luebbers (2021)  Canada; unspecified | Survey; n=18 | Determine physicians’ perceptions of the role of the dental hygienist in interprofessional collaboration. | Physicians | The survey addressed: personal experiences with a dental hygienist, dental hygienists’ roles working in collaboration with physicians, experiences with collaboration, benefits of working with dental hygienists, barriers, and demographics. |
| Macentee (1999)  Canada; RACF | Interviews  n=109; n/a | Contrast different human resource and organisational strategies for managing the delivery of oral health care to the elderly residents. | DHs, dentists, dental assistants, RACF administrators, directors of care, nurses, care-aids, clinic supervisor, physician | 109 people across 12 RACFs were interviewed. At the beginning of the study, the investigators assumed six strategies for delivering dental services to RACF and sought RACFs that could provide examples of each strategy. |
| Macentee (2006)  Canada; RACF | Literature review | Address the role and responsibility of dentistry in contributing to the health, dignity, and quality of life of frail elderly people living in RACFs | DHs, dentists, RACF staff | The author summarises the issues surrounding accessing dental care for frail elderly people and discusses strategies for addressing specific oral health conditions, as well as barriers to accessing care |
| First Author (Year)  Country; Setting | Study Methodology (groups); length  Total n; % with dementia | Purpose | Professional(s) Involved | Study Description |
| Marchini (2018)  USA; RACF | Cluster RCT pilot (3 groups); 6 months  n=81; 74% with dementia | Evaluate feasibility and gather initial data for a definitive study to test the clinical and microbiological effectiveness of a nursing facility customized oral hygiene protocol, intended to be delivered by dental hygienists and nursing personnel. | DHs, RACF staff | Group A (n=19; 74% with dementia): Control group.  Group B: (n=31; 65% with dementia): RACF staff received an educational program delivered by a DH. The DH conducted 1-hour interviews with a sample of staff, then a 1 hour tailored lecture aiming to address the specific issues raised by the staff in the interviews, and hands-on training. The DH visited the RACF every other week, where oral hygiene instructions were reinforced, and the DH brushed the resident’s teeth.  Group C (n=41; 84% with dementia): Educational program plus 1% chlorhexidine varnish applied monthly. |
| Morino (2014)  Japan; RACF | RCT (2 groups); 1-month  n=34; “most with cognitive disorders” | To better understand the role of the professional oral health care in improving geriatric oral health. | DHs | Intervention group (n=17): residents received professional oral health care once a week from two DHs.  Control group (n=17): usual routines were followed (self-care or  assisted by nursing staff) |
| Nakajima (2021)  Japan; palliative care | Surveys  n=515/n=74; n/a | Identify the challenges that DHs encountered when working with other professionals in a multidisciplinary team approach in palliative care for advanced cancer patients. | DHs, DH students | Study 1 (n=515): A questionnaire-based survey for DHs who belong to Japanese Society for Oral Care on oral care in palliative care settings.  Study 2: Involved two components (1) A cross-sectional analysis of the curriculum on palliative care at schools of oral health science in 10 universities in Japan. (2) A questionnaire-based survey on palliative care education at a school of oral health science at one of the 10 universities (n=74) |
| Niesten (2021)  The Netherlands; RACF | Interviews and stakeholder workshop  (2 groups)  n=93; n/a | Synthesise a framework of barriers and facilitators in the functional integration of oral health care into general health care for frail older adults at macro, meso, and micro levels. | DHs, dentists, nurses, RACF managers, dental clinic managers, GPs geriatricians | Interviews (n=41): Included 5 dentists, 3 DHs, 8 nurses, 4 GPs, 1 GP assistant, 6 geriatricians, 7 patients, 3 managers, 4 family caregivers. 24 worked for RACF patients.  Workshop (n=52): results and interpretations were discussed and refined |
| First Author (Year)  Country; Setting | Study Methodology (groups); length  Total n; % with dementia | Purpose | Professional(s) Involved | Study Description |
| Patterson-Norrie (2019)  Australia; RACF | Focus groups  (2 groups)  n= not stated; n/a | Explore the perceptions of residential aged care nursing and management staff regarding oral care, to develop strategies to improve the oral health of aged care residents. | Nursing staff, RACF management staff | Focus group 1: RACF management staff including a Nurse Unit Manager, Director of Nursing and CEO of the RACF group.  Focus group 2: nursing staff |
| Persson (2016)  Sweden; municipal organisations | Interviews  n=11; n/a | Explore how aspects of collaboration in an interprofessional and interorganizational intervention may lead to expansive learning. | DHs, nurses | Six DHs served non-clinically as oral health consultants in five Swedish municipal organizations. In order to explore aspects of how collaboration in an interprofessional and interorganizational intervention can lead to expansive learning, individual interviews were conducted, and reflection documents were collected and analysed. |
| Sangouam (2018)  Thailand; patients’ homes | Interviews  n=15 | To determine the roles of dental personnel in the Family Care Team Policy in lower–northern region, Thailand. | DHs, dentists | In-depth, semi-structured interviews were conducted with dental personnel in the Family Care Team Policy, which aimed at ensuring that people of all age groups are adequately cared for by the primary care unit closest to the public and linked to the physician from the district to the subdistrict hospital. |
| Seleskog (2018)  Sweden; RACF | Controlled clinical trial  (2 groups); 3 months  n=31; 68% with dementia | Trial a new oral healthcare educational programme and to evaluate the effects on residents’ oral health. | DHs, directors of nursing, nurses, nursing assistants | Intervention group (n=13): Nursing staff received weekly theoretical and hands-on guidance from two DHs on oral hygiene procedures and discussions on oral care routines. DHs provided personalised guidance and support for each resident once a week and personalised oral hygiene prescriptions for oral hygiene devices, procedures, or products.  Control group (n=18): usual routines were followed. The DHs could be contacted if needed. |
| Siegel (2017)  Australia; RACF | Systematic review | Review the content and effectiveness of interventions and implementation strategies used to improve or maintain the oral health of people with dementia or cognitive impairment. | DHs, dentists | 18 studies were included for review. Studies varied considerably in terms of size, scope, and focus. A number of methodological weaknesses were identified, and the overall quality of included studies was poor |
| First Author (Year)  Country; Setting | Study Methodology (groups); length  Total n; % with dementia | Purpose | Professional(s) Involved | Study Description |
| Simon (2019)  USA; tertiary care hospital | Case study  n=83; n/a | Describe the integration of a DH into the care teams of hospitalized patients with type 2 diabetes mellitus in a tertiary-care medical centre. | DHs, nurses | A DH provided care on three medical floors between 8-15 hours/week. They provided motivational interviewing, dental screening, and prophylaxis and had access to the patient’s electronic health record. |
| Smith (2017)  New Zealand; RACF | Interviews  n=20; n/a | Determine what challenges dentists face when providing domiciliary care to dependent older adults. | Dentists | The interviews explored the dentists’ observations from the New Zealand Older People’s Oral Health Survey, factors facilitating and hindering dependent older adults achieving or maintaining good oral health, their views on the current environment for dental care, and their opinions on how to provide care. |
| Sloane (2013)  USA; RACF | Pre-post study  (3 groups); 8 weeks  n=97; 85% with dementia | To develop and test a person-centred, evidence-based mouth care program in RACFs | DH, certified nursing assistants, psychologist | Two nursing assistants in each RACF acted as mouth-care aides, with on-site training by a DH and geriatric psychologist. Initial training was seminars on oral pathology, dementia care and individualised care planning skills. Training and supervision were provided daily for 2 weeks then decreased in frequency.  Specialised tools, 0.12% chlorhexidine rinse, and 1.1% sodium fluoride paste was provided. |
| Swanson Jaecks (2009)  USA; unspecified | Survey  n=103; n/a | Assess DH’s perceptions of their role in interdisciplinary collaboration, the barriers to effective collaboration, and communication skills needed to better participate in interdisciplinary collaboration. | DHs | A 45-item, quantitative survey instrument was completed by DHs recruited from 2 large dental hygiene meetings. The survey consisted of 5 sections: foundation questions, roles, barriers, communication skills and demographics |
| Theile (2016)  USA; unspecified | Case study and literature review | Explain the opportunity for the DH professional to embrace patient-centred care as an oral healthcare manager who can facilitate integration of oral and primary care in a variety of healthcare settings. | DHs | Based on an innovative model of collaboration between a college of dentistry and a college of nursing, an idea emerged among several faculty members for a new management method for realizing continuity and coordination of comprehensive patient care. |
| First Author (Year)  Country; Setting | Study Methodology (groups); length  Total n; % with dementia | Purpose | Professional(s) Involved | Study Description |
| Tynan (2018)  Australia; RACF | Pre-post study  (1 group); 6 months  n= 116; not stated | Describe the development and implementation of an integrated model of care at one regional at three rural RACFs and outline the lessons learnt. | OHTs, dentists, nurses, RACF staff | A quality improvement model was implemented across 4 RACFs. An OHT conducted oral health screens, developed personalised oral care plans, and referred residents to a tele-dentistry appointment with a dentist and the OHT where necessary. Each RACF allocated a nurse to the oral health portfolio and received training from the OHT to disseminate knowledge to other staff. |
| van der Putten (2013)  The Netherlands; RACF | Cluster RCT  (2 groups); 6 months  n=342; 48% with dementia | Assess the effectiveness of a supervised implementation of the “Oral health care Guideline for Older people in Long-term care Institutions”. | DHs, nurses | Intervention group (n=177): Supervised implementation of the Dutch “Oral health care (residential) care homes for elderly people” guideline. A nurse was appointed as the “ward oral health care organiser” (WOO). Training of the WOO involved theoretical and practical education according to the train-the-trainer concept. A DH made monitoring visits every 6 weeks for listing and resolving implementation problems and supporting the WOOs. |
| Volk (2020)  USA; RACF | Pre-post study  (1 group); 12 months  n=440; not stated | Discuss implementation of the quality improvement project. | DHs, nursing home staff | The Mouth Care without a Battle approach was implemented across 22 RACFs. DHs acted as coaches and were responsible for providing ongoing support to RACF staff, conducting spot-checks and any required assessments. They visited each RACF 3.3 per quarter on average. A RACF staff member was appointed as the program champion and led the team to implement the program. |
| Wallace (2013)  Australia; RACF | Descriptive Phenomenology n=67; n/a | Examine reflective journals to identify students’ experiences and perceived learning during a 12-week placement programme in RACFs. | DH students | Final year Dh students commenced a practicum in a RACF for 4 hours 1 day/week. Students were expected to initiate oral hygiene care for the residents and raise the profile of oral health with RACF staff and record their experiences in reflective journals. |
| Wallace (2016)  Australia; RACF | Pre-post study (1 group); 24 weeks  n=320; “some with cognitive impairments’ | Determine whether a qualified DH could improve oral health outcomes for residents living in residential aged care facilities. | DHs, RACF staff | The Senior Smiles model of care was implemented across 5 RACFs. The DH conducted oral health risk assessments for residents, developed individualized oral healthcare plans and provided referral pathways for residents who required more complex dental treatment. |
| First Author (Year)  Country; Setting | Study Methodology (groups); length  Total n; % with dementia | Purpose | Professional(s) Involved | Study Description |
| Weening-Vebree (2021)  The Netherlands; RACF | Questionnaires and focus groups  n=409/n=approx. 90; n/a | Explore attitudes, perceptions, and perceived barriers and facilitators of the daily oral health care performances among RACF staff. | Nursing staff, RACF managers, DHs. | Questionnaires: 409 nursing staff and 12 RACF managers completed a questionnaire aimed at gaining insight into the performance of dental care within the RACF.  Focus groups: 14 groups held with 6-8 people per group, across 21 RACFs. Participants included nursing staff, managers, and DHs. |
| Wintch (2014)  USA; RACF | Survey  n=38; n/a | Investigate knowledge and perceptions of RACF directors towards oral health of residents. | RACF executive directors | A survey was given to RACF executive directors to investigate perceptions and knowledge regarding oral health of RACF residents, protocol for provision of, and barriers to optimal oral health care, and support for employment of DHs in RACFs. |
| Yakiwchuk (2013)  Canada; RACF | Review article | Propose a multi targeted, multi strategy approach to champion change in RACFs that extends beyond the focus of clinical care along with strategies and tips for carrying it through. | DHs | The presented strategies and tips are organized within the dental hygiene paradigm of care, and described in the ADPIE format: Assessment, Diagnosis, Planning, Implementation, and Evaluation. They are drawn from DHs’ years of experience in caregiver engagement and training, resource development, and interprofessional collaboration within the RACF environment. |
| Yoneyama (2002)  Japan; RACF | RCT (2 groups);12 months  n=366; 68% with cognitive impairment | Investigate whether oral care lowers the frequency of pneumonia in RACF residents | DHs, dentists, nurses, caregivers | Intervention group (n=184): A DH or dentist provided professional care once a week across 11 RACFs. Nurses or caregivers cleaned residents’ teeth after each meal.  Control group (n=182): Usual routines were followed (no staff assistance) across 11 RACFs |
| Yoon (2012)  Canada; RACF | Focus groups  n=28; n/a | To explore perspectives regarding oral care held by in RACFs and to understand how their perspectives impact activities and processes involved in the delivery of oral care. | DHs, nurses, speech-language pathologists, directors of care, personal support workers | Separate focus groups for each profession were held (DHs n=4, all others n=6). Focus groups were facilitated by the researcher using a non-directive interview technique with open-ended questions. |
| Abbreviations: RACF = residential aged care facility, DH = dental hygienist, DHT = dental hygienist-therapist, OHT = oral health therapist, GP = general practitioner, MMSE = Mini-Mental State Examination. | | | | |
