# Supplementary material for: Integrating Dental Professionals Into Aged Care With Focus on Australia: A Scoping Review
Source: Gerodontology. 2025 Jan 12;42(2):147–64. doi: 10.1111/ger.12784 (PMC12106945; doi:10.1111/ger.12784)
Supplement: Supplementary file 2 — Table S2. [file GER-42-147-s002.docx]

| **Summary of Studies Reporting on Feasibility** | | | | | | | | |
| --- | --- | --- | --- | --- | --- | --- | --- | --- |
| First Author (Year) | Area of Focus | | | | | | | |
|  | Acceptability | Demand | Implementation | Practicality | Adaptation | Integration | Expansion | Limited Efficacy |
| Aagaard (2020) | x^a^ | x | +^b^ | x | x | x | x | x |
| Amerine (2014) | x | x | x | -^c^ | x | x | x | x |
| Brondani (2011) | + | x | x | x | x | x | x | x |
| Compton (2013) | +/-^d^ | x | - | x | x | x | x | x |
| Hearn (2016) | x | + | x | x | x | x | x | x |
| Hopcraft (2011) | x | x | x | x | x | x | x | + |
| Huynh (2017) | x | x | x | + | x | x | x | x |
| Kullberg (2009) | x | x | + | x | x | x | x | x |
| Kullberg (2010) | + | + | x | x | x | x | x | x |
| Macentee (1999) | x | + | x | x | x | x | x | x |
| Morino (2014) | x | x | x | x | x | + | x | x |
| Niesten (2021) | x | x | x | x | x | x | x | x |
| Patterson-Norrie (2019) | x | + | x | x | x | + | x | x |
| Seleskog (2018) | x | x | x | x | x | x | x | - |
| Sloane (2013) | + | x | +/- | + | x | x | x | x |
| Smith (2017) | x | x | x | x | x | + | x | x |
| Tynan (2018) | + | x | x | + | x | x | x | x |
| van der Putten (2013) | x | x | - | x | x | x | x | x |
| Volk (2020) | x | x | x | x | x | +/- | x | x |
| Wallace (2013) | x | x | +/- | x | x | x | x | x |
| Wallace (2016) | x | x | x | x | x | - | x | x |
| Weening-Vebree (2021) | x | + | x | x | x | x | x | x |
| Wintch (2014) | x | + | x | x | x | +/- | x | x |
| Total Number of Studies | 4 | 6 | 6 | 4 | 0 | 6 | 0 | 1 |
| ^a^ Not addressed  ^b^ Positive outcome  ^c^ Negative outcome  ^d^ Mixed Outcome | | | | | | | | |
